# Matrix metalloproteinase-9 is elevated and related to interleukin-17 and psychological stress in male infertility: A cross-sectional study

**DOI:** 10.18502/ijrm.v19i4.9059

**Published:** 2021-04-22

**Authors:** Ann Prasad Mary, Hanumanthappa Nandeesha, Dasari Papa, Thyagaraju Chitra, Rajesh Nachiappa Ganesh, Vikas Menon

**Affiliations:** ^1^Biochemistry Department, Jawaharlal Institute of Postgraduate Medical Education and Research (JIPMER), Puducherry, India.; ^2^Department of Obstetrics and Gynecology, Jawaharlal Institute of Postgraduate Medical Education and Research (JIPMER), Puducherry, India.; ^3^Department of Pathology, Jawaharlal Institute of Postgraduate Medical Education and Research (JIPMER), Puducherry, India.; ^4^Department of Psychiatry, Jawaharlal Institute of Postgraduate Medical Education and Research (JIPMER), Puducherry, India.

**Keywords:** Infertility, Interleukins, Peptide hydrolase.

## Abstract

**Background:**

Matrix metalloproteinase-9 (MMP-9), interleukin-17 (IL-17) and psychological stress are known to play a role in the pathogenesis of male infertility.

**Objective:**

To assess the association of MMP-9 with IL-17 and psychological stress in infertile men.

**Materials and Methods:**

In this cross-sectional study, 39 men with infertility diagnosed based on semen analysis and 39 subjects with normal semen analysis were included in the study. MMP-9 and IL-17 were estimated in both groups by ELISA. Perceived stress scale was used to assess psychological stress in controls and cases.

**Results:**

In infertile cases, MMP-9 and IL-17 were significantly increased when compared with controls (p = 0.046, p = 0.041 respectively). A significant association of MMP-9 was observed with IL-17 (r = 0.335, p = 0.037) and perceived stress scale (r = 0.329, p = 0.041).

**Conclusion:**

IL-17 and stress increase MMP- 9 levels in infertile men.

## 1. Introduction

The prevalence of primary infertility is increasing worldwide including in India, where around 23% of cases have been attributed to male factors (1). A 13-yr study in south India found that the sperm count, sperm motility, and morphology decreased by 30.3%, 22.92% and 51.25% respectively (2). Among various factors, stress and inflammation are widely associated with the pathogenesis of infertility.

Interleukin-17 (IL-17) is a pro-inflammatory cytokine, that appearsto be involved in the maintenance of testicular immunity and spermatogenesis (3). Higher levels of IL-17 have been reported in patients with azoospermia and increased expression was found to be associated with sperm damage (3). A previous study by Qian and colleagues has found a negative relationship between IL-17 and sperm motility and apositive relationship with sperm mortality (4).

Matrix metalloproteinases (MMPs) are zinc-dependent proteinases, which have beenreported to be involved in the sperm generation and differentiation (5). The MMP-9 expression in seminal plasma has been reported in men with normal sperm count (6). A previous study has reported an increase in MMP-9 activity in men with low sperm count and found a significant correlation with sperm concentration (7).

Psychological stress is known to affectmale fertility and it was demonstrated to reduce sperm quality and paternity (8). It was found that semen parameters in men decreaseduring thein-vitro fertilization treatment of their spouses whichattributed to psychological stress (9). Since MMP-9 levels are influenced by stress and inflammation, the present study was designed to investigate the MMP-9 levels in seminal plasma and its associationwith IL-17 and psychological stress in male infertility.

## 2. Materials and Methods

In this cross-sectional study, 78 men (39 infertile and 39 men with normal semen analysis), who referred to the Jawaharlal Institute of Postgraduate Medical Education and Research (JIPMER) between January 2018 and February 2019 for infertility treatment of their spouses were enrolled. Based on the World Health Organization criteria (10), infertility was diagnosed as sperm count ≤ 15 million/ ml, or sperm motility ≤ 40%, or sperm morphology of ≤ 4%. Men with major medical disorders, psychiatric disorders, and those taking anti-inflammatory or anti-psychotic drugs were excluded from the study.

### Sample size calculation

The sample size was estimated based on the differences in the mean level of MMP-9 between the control and the cases. The expected difference in the mean MMP-9 levels was found to be 3.08 ng/ml between the controls and the cases (11). The sample size was estimated at a power of 90% and at 5% significance level.

### Study procedure

Semen sampleswere obtained from infertility cases and controls after three days of abstinence from sexual activity. Seminal plasma was collected after the centrifugation of the semen samples and stored at –400 C until the estimation of MMP-9 and IL-17. Psychological stress was analyzed using the Perceived Stress Scale (PSS) (12). Although several tools are available for assessing the psychological stress, PSS is widely used to in the Indian subcontinent. Moreover, the PSS was validated by the psychiatry department for psychological stress assessment.

### Perceived stress scale (PSS)

The PSS is a classic stress assessment instrument. The questions on this scale are about the individual's feelings and thoughts over the past one month. In each case, the individual will be asked to indicate how often they feel or thinks of a certain way. Scores are determined using a few directions as prescribed by the developer. The total score in PSS can range from 0 to 40 (Low stress: 0-13; moderate stress: 14-26; and high stress: 27-40) (12).

### IL-17 and MMP-9 estimation

Seminal IL-17 and seminal MMP-9 were estimated by ELISA using the Human IL-17 ELISA kits (Diaclone, France) and the Human MMP-9 ELISA kits (Elabscience, USA).

### Ethical considerations

The present study was approved by the Institution Ethics Committee for Human Studies (Ref: JIP/IEC/2017/0343). In addition, written informed consent was obtained from all the subjects before the study.

### Statistical analysis 

Normally distributed data are represented as mean and standard deviation while the non- normally distributed data are represented as median and interquartile range. Categorical data are represented as frequency and percentage. The normality of continuous data was detected using the Kolmogorov-Smirnov test and independent *t* test and Mann-Whitney U- test were used to compare the differences in semen parameters and biochemical parameters between the controls and cases and subjects with moderate and severe psychological stress. Additionally, the correlation between the variables was analyzed using Spearman's rank correlation test. P < 0.05 was considered significant. The results were analyzed using the Statistical Package for the Social Sciences, version 16.0, SPSS Inc, Chicago, Illinois, USA (SPSS).

## 3. Results

Table I shows the general characteristics, duration of infertility and semen analysis parameters in controls and infertility cases. IL-17 and MMP-9 were significantly increased and sperm count, motility and morphology were significantly decreasedin cases indicating the presence of inflammation and abnormal semen parameters in men with infertility.

Table II presents the association of IL-17 and MMP-9 with semen analysis parameters, PSS, age and the duration of infertility in infertile cases. MMP-9 was positively associated with IL-17 and PSS, suggesting that increase in stress and inflammation increases the MMP-9 levels. IL-17 was significantly correlated with sperm morphology suggesting inflammation can alter semen parameters in infertility.

Moreover, sperm motility was negatively correlated with age and the duration of infertility suggesting an inverse relationship of sperm motility with anincrease in age and duration of infertility.

Table III shows the effect of psychological stress on semen parameters, IL-17 and MMP-9 in infertility cases. There were no significant differences in IL-17, MMP-9, and semen analysis parameters among infertile men with severe stress compared to those with moderate stress.

**Table 1:** General characteristics, duration of infertility and semen analysis parameters in controls and infertile cases


**Parameters**	**Controls (n = 39)**	**Infertile cases (n = 39)**	**P-value**
Age (yr)*	30.2 ± 4.4	31.7 ± 4.6	0.152${}^{a}$
Body mass index (kg/m ${}^{2}$)*	24.42 ± 3.23	25.49 ± 2.62	0.06${}^{a}$
Duration of infertility (yr)*	3.38 ± 1.77	3.18 ± 1.52	0.585${}^{a}$
Sperm count (millions)*	64.12 ± 38.14	5.9 ± 3.8	< 0.001${}^{b}$
Sperm motility (%)**	45 (6–126)	6 (0–35)	< 0.001${}^{b}$
Sperm morphology (%)**	10 (2–70)	2 (1–6)	< 0.001${}^{b}$
**Abnormalities of semen parameters**			
Oligozoospermia***	3 (7)	39 (100)	
Asthenozoospermia***	4 (10)	33 (85)	
Altered sperm morphology***	33 (85)	39 (100)	
Perceived stress scale*	21.79 ± 6.51	23.0 ± 6.50	0.416${}^{a}$
Interleukin-17 (ng/L)**	26.54 (1.92–67.44)	35.92 (6.64–143.6)	0.041${}^{b}$
Matrix metalloproteinase-9 (µg/L)**	16.18 (4.29–140.40)	31.18 (9.06–330.43)	0.046${}^{b}$
* Data presented as Mean ± SD, **Data presented as Median (Range), ***Data presented as n (%), a: Independent *t* test, b: Mann–Whitney U- test, ${}^{a}$Independent *t* test, ${}^{b}$Mann–Whitney U-test			

**Table 2:** Correlation of interleukin-17 and matrix metalloproteinase-9 with semen analysis parameters, perceived stress scale, age and duration of infertility in infertility cases (n = 39)


	**Interleukin-17**		**Matrix metalloproteinase-9**	
<brow>-2</erow> **Parameters**	**R**	**P**	**R**	**P-value**
**Age **	–0.040	0.808	0.157	0.339
**Duration of infertility**	–0.089	0.588	0.199	0.224
**Sperm count **	0.202	0.218	–0.091	0.583
**Sperm motility **	0.250	0.125	–0.192	0.241
**Sperm morphology **	0.340	0.034	–0.086	0.601
**Perceived stress scale**	–0.024	0.884	0.329	0.041
**Interleukin-17**	–	–	0.335	0.037
R = Correlation coefficient, Spearman correlation test				

**Table 3:** Effect of psychological stress on semen parameters, interleukin-17 and matrix metalloproteinase-9 in men who attended infertility clinic (n = 78)


**Parameters**	**Moderate stress (PSS: 13–26) (n = 59)**	**Severe stress (PSS: 27-40) (n = 19)**	**P-value**
**Sperm count (millions)**	10.5 (1–131.2)	7 (1–85.2)	0.139
**Sperm motility (%)**	32 (1–126.1)	12 (0–53)	0.086
**Sperm morphology (%)**	5 (1–70)	2 (1–14)	0.104
**Interleukin-17**	30.14 (1.9–143.64)	20.2 (5.98–94)	0.120
**Matrix metalloproteinase-9**	16.18 (4.29–236.76)	52.91 (11.30–330.43)	0.059
PSS: Perceived stress scale, data presented as non-normal by Kolmogorov–Smirnov test and analyzed by Mann–Whitney U-test			

## 4. Discussion

In the present study, IL-17 and MMP-9 were significantly elevated in infertility cases compared to controls. MMP-9 was positively correlated with PSS and IL-17 in subjects with infertility.

Inflammation is one of the factors that play a role in the etiopathogenesis of infertility and several studies have revealed alteration in cytokine levels in subjects with infertility (13). Previous studies have reported high IL-17 levels in semen samples with low-activity sperms (4, 14). These studies also observed that IL-17 did not influence sperm density and morphology (4). In the present study IL-17 was significantly increased in infertility cases when compared with controls. Also IL-17 was significantly associated with sperm morphology indicating that inflammation can alter sperm structure there by reducing the process of fertilization. These findings are in agreement with previous studies that demonstrated elevated IL-17 levels in subjects with azoospermia (3).

Matrix metalloproteinase-9 plays a role in liquefaction of semen and its levels are altered in infertility (5). Elevated seminal MMP-9 levels were demonstrated by previous investigators in subjects with low sperm concentration (11). In the present study MMP-9 was significantly increased in infertility cases when compared with controls (p = 0.046). We did not observe any significant association of MMP-9 with semen parameters which were similar to the findings reported by Tentes and colleagues (11). We found a positive association of MMP-9 with IL-17 suggesting that inflammation can increase MMP-9 levels in subjects with infertility. Apart from semen liquefaction, it has been speculated that MMP-9 can cause acrosomal damage and sperm apoptosis, which might diminish the chances of fertilization (5, 15).

Several experimental and clinical studies have demonstrated the association of psychological stress with abnormal semen parameters and reduced paternity (8, 16, 17). Wirleitner and colleagues have shown an inverse relationship between psychological stress and thequality of semen (9). In the present study, psychological stress was assessed using the PSS. We observed that majority of the cases had moderate stress followed by severe stress and mild stress. There were no significant differences in stress levels between cases and controls suggesting that irrespective of the causes, male partners are under stress due to infertility. MMP-9 was positively associated with PSS suggesting stress can increase MMP-9 levels in these subjects. When male subjects were divided into moderate and severe stress groups based on the PSS score, we found that MMP-9 level was elevated and the sperm count, motility and morphology were reduced in subjects with severe stress, but it was not significant. This can be attributed to the small sample size in severe stress group compared to those with moderate stress group.

Activation of the HPA axis (hypothalamic-pituitary-adrenal) plays a key role in neuro-endocrine response to stress. Acute stress leads to suppression of theHPG axis through inhibition of gonadotrophin-releasing hormone (GnRH) (18). We hypothesize that psychological stress leads to an imbalance of endocrine mechanismswhich may contribute to abnormal semen parameters leading to infertility.

Assessing the levels of MMP-9 and IL-17 can not indicate the effect of psychological stress on sperm parameters. Our hypothesis was psychological stress increases inflammation which might be associated with sperm abnormalities. In the present study, MMP-9 was positively associated with PSS and IL-17. Hence we conclude that stress and inflammation may increase MMP-9 which in turn might cause sperm abnormalities.

## 5. Conclusion

The present study concludes that MMP-9 and IL-17 are increased in men with abnormal semen parameters. The association of MMP-9 with PSS and IL-17 suggests that MMP-9 levels are affected by stress and inflammation which in turn leads to abnormalities in sperm count, motility and morphology. Further studies are needed to investigate whether a reduction in inflammation and stress could reduce MMP-9 and normalizes the semen parameters in men with infertility.

##  Conflict of Interest

The authors report no conflict of interest.
